# Development and validation of a web-based predictive model for preoperative diagnosis of localized colorectal cancer and colorectal adenoma

**DOI:** 10.3389/fonc.2023.1199868

**Published:** 2023-08-17

**Authors:** Yan Lu, Haoyang Guo, Jinwen Jiang

**Affiliations:** Clinical Laboratory, DongYang People’s Hospital, Dongyang, Zhejiang, China

**Keywords:** colorectal cancer, colorectal adenoma, predictive model, preoperative diagnosis, dynamic nomogram

## Abstract

**Background:**

Localized colorectal cancer (LCC) has obscure clinical signs, which are difficult to distinguish from colorectal adenoma (CA). This study aimed to develop and validate a web-based predictive model for preoperative diagnosis of LCC and CA.

**Methods:**

We conducted a retrospective study that included data from 500 patients with LCC and 980 patients with CA who were admitted to Dongyang People’s Hospital between November 2012 and June 2022. Patients were randomly divided into the training (n=1036) and validation (n=444) cohorts. Univariate logistic regression, least absolute shrinkage and selection operator regression, and multivariate logistic regression were used to select the variables for predictive models. The area under the curve (AUC), calibration curve, decision curve analysis (DCA), and clinical impact curve (CIC) were used to evaluate the performance of the model.

**Results:**

The web-based predictive model was developed, including nine independent risk factors: age, sex, drinking history, white blood cell count, lymphocyte count, red blood cell distribution width, albumin, carcinoembryonic antigen, and fecal occult blood test. The AUC of the prediction model in the training and validation cohorts was 0.910 (0.892–0.929) and 0.894 (0.862–0.925), respectively. The calibration curve showed good consistency between the outcome predicted by the model and the actual diagnosis. DCA and CIC showed that the predictive model had a good clinical application value.

**Conclusion:**

This study first developed a web-based preoperative prediction model, which can discriminate LCC from CA and can be used to quantitatively assess the risks and benefits in clinical practice.

## 1 Introduction

Colorectal cancer (CRC) is the second leading cause of cancer-related death (1). However, for patients with localized colorectal cancer (LCC), the 5-year survival rate after timely surgical treatment can reach 90% (2). Generally, endoscopy predicts potential malignant tumors based on the size and shape of colorectal tumors and ultimately guides tumor treatment (3). LCC and colorectal adenoma (CA) are local lesions that require different treatment approaches. Patients with LCC should undergo laparoscopic or open surgery as soon as it is practicable (4) and may require chemotherapy and adjuvant radiation before surgery (5). On the contrary, patients with CA can be treated using selective endoscopic removal based on their preference (6). However, LCC has obscure clinical signs, and is difficult to distinguish from CA, which depends on biopsy and pathological evaluation (7).

Pathological diagnosis is the gold standard for differentiating between benign and malignant colorectal tumors. However, an endoscopic biopsy is an invasive examination that can lead to complications, such as bleeding, perforation, and infection; thus, it is limited to the patient’s willingness and compliance (8). Due to the advancements in molecular diagnostic technology, DNA (9) and microRNA (10) are now being used as CRC biomarkers. However, they are expensive with unstable diagnostic performance limiting their use in clinical settings. Therefore, fecal occult blood test (FOBT) (11) and serum carcinoembryonic antigen (CEA) (12) detection are preferred as CRC biomarkers because of their accessibility and affordability. However, for CRC, particularly in the early stage, a single detection biomarker has limited sensitivity and a high probability of misdiagnosis. To select the appropriate treatment method and reduce the rate of misdiagnosis of LCC, it is crucial to create a diagnostic prediction model with excellent diagnostic performance using readily accessible and affordable markers.

In previous studies, there are many potential predictive biomarkers for CRC preoperative diagnosis models, such as platelet-related parameters (13), red blood cell distribution width (RDW) (14), and hemoglobin (15). However, some of those studies had a small sample size (16) and almost all of them included patients with advanced CRC (17, 18). To our knowledge, patients with advanced CRC present with more pronounced clinical symptoms; as such, less sensitive indicators have an exaggerated role in the prediction model, resulting in the reduced diagnostic performance of the LCC prediction model.

In this single-center retrospective study, we used clinical and laboratory data of patients from 2012 to 2022 to develop and validate the first web-based predictive model for preoperative diagnosis of LCC and CA.

## 2 Materials and methods

The design and reporting of this study were guided by transparent reporting of a multivariable prediction model for individual prognosis or diagnosis (TRIPOD) (19).

### 2.1 Study population

**Figure 1:**
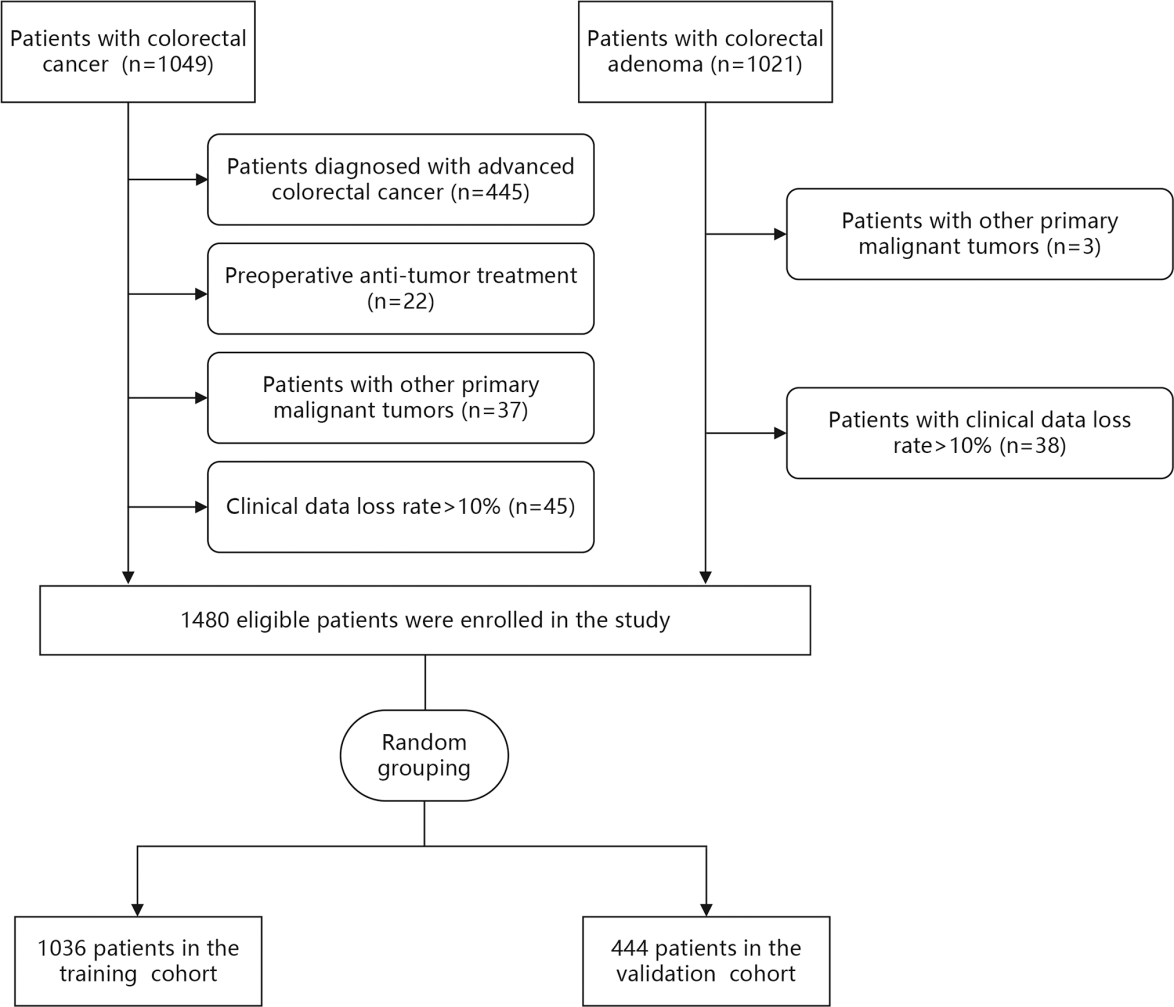
Flow chart of the study.

Demographic and clinical data of 2070 patients were collected from the medical records at the Dongyang People’s Hospital between November 2012 and June 2022. The inclusion criteria were adult patients (≥ 18 years) who were diagnosed with CRC or CA using histopathology. The exclusion criteria were (1): preoperative anti-tumor treatment (2), patients diagnosed with advanced CRC (Tumor-Node-Metastasis [TNM] stage III-IV) according to TNM staging of the eighth edition of the American Joint Committee on Cancer (3), patients with other primary malignant tumors, and (4) patients with clinical data loss rate >10%. According to the inclusion and exclusion criteria, 500 patients with LCC and 980 patients with CA were finally included in this study (**Figure 1**).

The study was approved by the ethics committee of Dongyang People’s Hospital. The data analysis was anonymous. Informed consent was waived since this is a retrospective study and the diagnosis as well as treatment of the patients were not affected.

### 2.2 Data collection

Preoperative data of eligible patients were extracted, including age, sex, routine blood parameters, FOBT, serum albumin, serum glucose, and tumor biomarkers. Routine blood parameters included white blood cell (WBC), neutrophil, and lymphocyte counts, as well as hemoglobin, RDW, platelet count, and mean platelet volume. Selected tumor biomarkers were CEA and carbohydrate antigen 199. FOBT-positive patients were defined as those with a positive immunochemical test and guaiac-based test ≥ 1+. When the proportion of missing values in the variables was less than 10%, the missing data were filled using multiple imputations in the two cohorts respectively (20). For outliers, which were defined as values other than 1-99% in continuous variables winsorized by 1% on both sides (21), artificial discrimination was used in categorical variables. In addition, data on smoking, drinking, diabetes, and hypertension statuses of the eligible patients were collected. A history of smoking or drinking was noted if smoking or the use of alcohol was reported in a patient’s medical record.

### 2.3 Statistical analysis

Included patients were randomly divided into the training (70%) and validation (30%) cohorts. Data were analyzed using Stata version 14.0 (Stata Corp LP, College Station, TX, USA) and R software version 4.1.0. Continuous variables are expressed as means and standard deviations or medians and interquartile intervals as appropriate. A Wilcox test or Student’s t-test was used to assess between-group differences. Categorical variables were compared using the chi-square test and are presented as quantities (percentages).

Data from the training cohort were used for the development of the prediction model. Univariable logistic regression analysis was used preliminarily to screen candidate variables. The least absolute shrinkage and selection operator (LASSO) regression analysis was then used to remove collinear independent variables to prevent overfitting (22). Subsequently, indicators with coefficients that were not zero were included in a multivariable logistic regression to complete the final selection of variables. Variables with statistical significance (*P<*0.05) were included in the prediction model. The prediction probability of the model was calculated using the following formula:


$$\mathrm{Pr}obability=\frac{1}{1+\exp(-(\beta 0+\beta 1\chi 1+\beta 2\chi 2+\text{...}+\beta n\chi n))}$$

A web-based prediction model was developed based on the “DynNom” and “rsconnect” packages in R software (23). The prediction probability can be automatically calculated after inputting the expression of each variable.

The performance of the model in the training and validation cohort was assessed. After data were normalized, the discriminative ability was expressed using the area under the receiver operating characteristic (ROC) curve (AUC). The Youden index was used to determine the best cut-off value, and the corresponding sensitivity, specificity, accuracy, positive prediction value (PPV), negative prediction value (NPV), positive likelihood ratio (PLR), as well as negative effect ratio (NLR), were calculated. To examine the consistency between the actual risk of LCC and the probability predicted using the new model, calibration curves were plotted and the Hosmer–Lemeshow goodness of fit test was performed. The clinical utility of the decision curve analysis (DCA) and clinical impact curve (CIC) was used to demonstrate the clinical utility of the prediction model (24).

## 3 Results

### 3.1 Patient characteristics

Demographic and clinical data were extracted from medical records of 2070 patients between November 2012 and June 2022. After data were excluded based on our criteria, records of 1480 patients (LCC: n=500 and CA: n=980) were finally included in this study (**Figure 1**). Among LCC patients, five (1.0%), 161 (32.2%), and 334 (66.8%) patients had TNM stage 0, I, and II disease, respectively. The patients were then randomly divided into the training (n=1036) and validation (n=444) cohorts at a ratio of 7:3. There was no statistical difference between the training and validation cohorts in each variable (**Table 1**).

**Table 1:** Preoperative baseline characteristics of patients in the training and validation cohorts.

Variables	Training Cohort		Validation Cohort		*P*
	CA Group (n = 684)	LCC Group (n = 352)	CA Group (n = 296)	LCC Group (n = 148)	
Age, years	58.4 ± 11.3	65.6 ± 12.1	57.1 ± 11.3	65.1 ± 10.8	0.132
Male, n (%) Female, n (%)	485 (70.9) 199 (29.1)	193 (54.8) 159 (45.2)	214 (72.3) 82 (27.7)	81 (54.7) 67 (45.3)	0.711
Smoking history, n (%)	246 (36.0)	105 (29.8)	120 (40.5)	43 (29.1)	0.294
Drinking history, n (%)	225 (32.9)	139 (39.5)	107 (36.1)	45 (30.4)	0.739
Comorbidities, n (%)					
Hypertension	190 (27.8)	134 (38.1)	70 (23.6)	54 (36.5)	0.199
Diabetes	35 (5.1)	27 (7.7)	19 (6.4)	17 (11.5)	0.132
Laboratory test					
White blood cell count, 10 ^9^/L	5.6 (4.8–6.7)	6.1 (4.9–7.4)	5.7 (4.8–6.7)	6.2 (5.0–7.6)	0.616
Neutrophil count, 10 ^9^/L	3.4 (2.8–4.3)	3.9 (2.9–5.0)	3.4 (2.8–4.2)	3.9 (3.0–5.1)	0.905
Lymphocyte count, 10 ^9^/L	1.7 (1.4–2.0)	1.5 (1.2–1.8)	1.7 (1.4–2.1)	1.6 (1.2–1.9)	0.083
Hemoglobin, g/L	146 (136–156)	129 (114–141)	147 (136–157)	126 (114–140)	0.866
Red blood cell distribution width, %	12.5 (12.2–13.0)	13 (12.5–14)	12.6 (12.2–13.1)	13.2 (12.5–14)	0.414
Platelet count, 10 ^9^/L	215 (180.5–253)	238 (195–288)	215 (183–255)	237.5 (186–289.5)	0.909
Mean platelet volume, fL	10.5 (10–11.2)	10.2 (9.6–10.8)	10.6 (10–11.3)	10 (9.4–10.8)	0.944
Glucose, mg/dL	5.15 (4.8–5.6)	5.1 (4.7–5.6)	5.1 (4.8–5.6)	5.2 (4.8–6.1)	0.749
Albumin, g/L	44.3 (42.4–46.1)	40.5 (36.6–43.4)	44.1 (42.3–46.2)	40.6 (36.1–43.6)	0.902
Carcinoembryonic antigen, ng/mL	2.2 (1.5–3.3)	3.7 (2.2–6.9)	2.2 (1.5–3.3)	3.3 (2.0–5.3)	0.212
Carbohydrate antigen 199, U/mL	9.6 (6.5–14.6)	10.7 (6.4–17.6)	10.1 (6.4–14.5)	10.2 (6.4–17.6)	0.747
Fecal occult blood test, n (%)	153 (22.4)	279 (79.3)	77 (26.0)	120 (81.1)	0.341
TNM stage, n (%)					0.867
Stage 0		4 (1.1)		1 (0.7)	
Stage I		112 (31.8)		49 (33.1)	
Stage II		236 (67.1)		98 (66.2)	

LCC, localized colorectal cancer; CA, colorectal adenoma; TNM, tumor-node-metastasis.

### 3.2 Variable selection and development of a web-based predictive model

As shown in **Table 2**, in the training cohort, there were significant differences in 16 clinical parameters between LCC and CA groups using univariable logistic regression. Subsequently, 15 potential predictors without multicollinearity were identified using LASSO regression analysis (**Table 2**, **Supplementary Figure 1**). Finally, age, sex, drinking history, WBC, lymphocyte count, RDW, albumin, CEA, and FOBT were considered independent predictors of LCC in patients according to the results of multivariate logistic regression (**Table 3**). The probability of LCC can be calculated according to the following formula: Probability (LCC) = 1/(1 + exp (− (-1.703 + 0.024 × age -1.035 × sex (male=1, female=0) + 0.482 × drinking history (yes=1, no=0) + 0.215 × WBC - 0.694 × lymphocyte count + 0.334 × RDW - 0.131 × albumin + 0.203 × CEA + 2.357 × FOBT (positive=1, negative=0)))).

**Table 2:** Univariate logistic analysis and LASSO regression analysis in the training cohort.

Variables	Univariable logistic analysis		LASSO regression analysis
	OR (95% CI)	*P*	Lambda.min = 0.0023
Age	1.06 (1.04–1.07)	<0.001	0.0248
Sex (male=1, female=0)	0.50 (0.38–0.65)	<0.001	-0.6563
Smoking history (yes=1, no=0)	0.76 (0.57–1.00)	0.048	-0.0539
Drinking history (yes=1, no=0)	1.33 (1.02–1.74)	0.035	0.4375
Hypertension (yes=1, no=0)	1.60 (1.22–2.10)	0.001	0.0425
Diabetes (yes=1, no=0)	1.54 (0.92–2.59)	0.103	/
White blood cell count	1.15 (1.08–1.23)	<0.001	0.1896
Neutrophil count	1.27 (1.17–1.38)	<0.001	0
Lymphocyte count	0.49 (0.38–0.63)	<0.001	-0.6331
Hemoglobin	0.95 (0.94–0.96)	<0.001	-0.0117
Red blood cell distribution width	1.85 (1.61–2.14)	<0.001	0.1904
Platelet count	1.01 (1.00–1.01)	<0.001	0.0029
Mean platelet volume	0.63 (0.55–0.72)	<0.001	-0.0030
Glucose	1.02 (0.95–1.09)	0.552	/
Albumin	0.78 (0.75–0.81)	<0.001	-0.1077
Carcinoembryonic antigen	1.32 (1.24–1.40)	<0.001	0.1713
Carbohydrate antigen 199	1.02 (1.01–1.03)	<0.001	0.0017
Fecal occult blood test (positive=1, negative=0)	13.26 (9.69–18.16)	<0.001	2.2308

CI, confidence interval; OR, odds ratio; LASSO, least absolute shrinkage and selection operator.

**Table 3:** Multivariate logistic analysis in the training cohort.

Variables		Multivariate logistic analysis				
	β	OR (95% CI)	*P*	β	OR (95% CI)	*P*
Age	0.026	1.03 (1.01–1.05)	0.006	0.024	1.02 (1.01–1.04)	0.006
Sex (male=1, female=0)	-0.734	0.48 (0.29–0.79)	0.004	-1.035	0.35 (0.23–0.54)	<0.001
Smoking history (yes=1, no=0)	-0.137	0.87 (0.52–1.46)	0.602			
Drinking history (yes=1, no=0)	0.532	1.70 (1.07–2.70)	0.024	0.482	1.62 (1.07–2.45)	0.023
Hypertension (yes=1, no=0)	0.073	1.08 (0.71–1.62)	0.726			
White blood cell count	0.207	1.23 (1.09–1.38)	0.001	0.215	1.24 (1.11–1.39)	<0.001
Lymphocyte count	-0.701	0.50 (0.33–0.74)	0.001	-0.694	0.50 (0.34–0.74)	0.001
Hemoglobin	-0.011	0.99 (0.98–1.00)	0.083			
Red blood cell distribution width	0.215	1.24 (1.02–1.50)	0.027	0.334	1.40 (1.18–1.65)	<0.001
Platelet count	0.003	1.00 (1.00–1.01)	0.069			
Mean platelet volume	-0.129	0.88 (0.71–1.09)	0.231			
Albumin	-0.110	0.90 (0.85–0.95)	<0.001	-0.131	0.88 (0.83–0.92)	<0.001
Carcinoembryonic antigen	0.202	1.22 (1.13–1.32)	<0.001	0.203	1.22 (1.14–1.32)	<0.001
Carbohydrate antigen 19-9	0.002	1.00 (0.99–1.01)	0.719			
Fecal occult blood test (positive=1, negative=0)	2.287	9.84 (6.71–14.43)	<0.001	2.357	10.56 (7.24–15.42)	<0.001
Intercept	-0.054	/	/	-1.703		

CI, confidence interval; OR, odds ratio.

**Figure 2:**
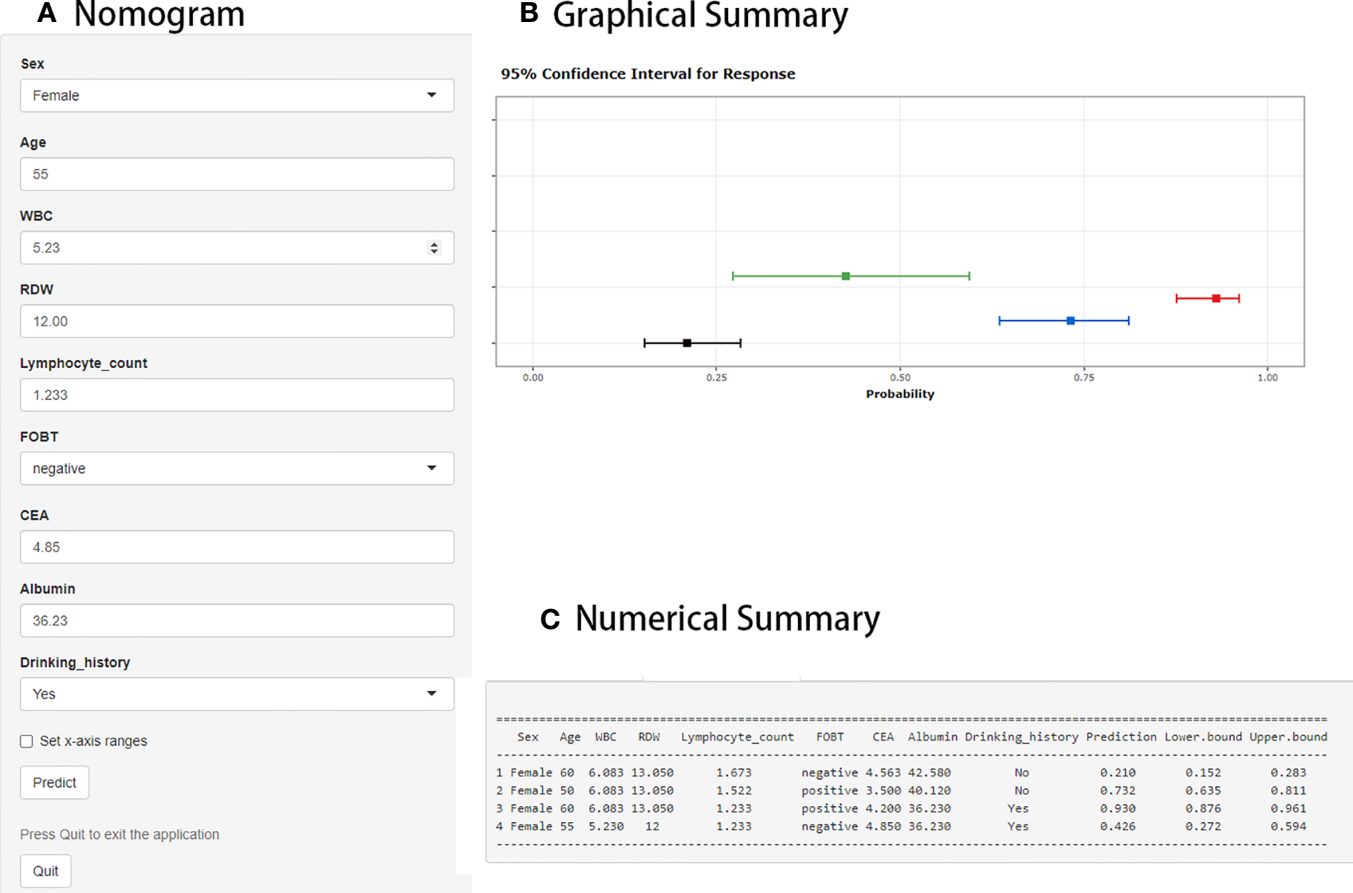
Web interface for distinguishing localized colorectal cancer (LCC) from colorectal adenoma (CA). **(A)** The area where the expression of each item can be adjusted by the user. **(B)** Graphical summary of the LCC probability and 95% confidence interval (CI) predicted by the prediction model. **(C)** Numerical summary of LCC probability and 95% CI.

The web-based dynamic prediction model developed using the selected variables can be used through the following link: https://ly11219.shinyapps.io/dynnomapp/.The interface of this webpage is shown in **Figure 2**. **Figure 2A** displays the input nomograph interface, in which users can adjust the expression of each item. **Figure 2B** shows a graphical summary of the LCC probability and 95% confidence interval (CI) predicted for the three patients according to the nomogram. The page also provides a numerical summary, shown in **Figure 2C**.

### 3.3 Evaluation of the performance of the prediction model

**Figure 3:**
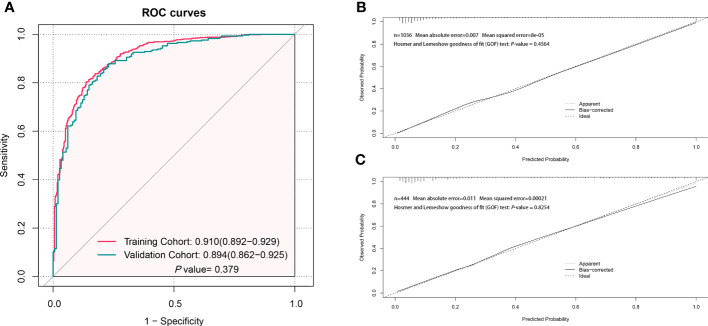
Evaluation of the performance of the prediction model to distinguish localized colorectal cancer (LCC) and colorectal adenoma (CA). **(A)** The receiver operating characteristic (ROC) curve of the prediction model in the training and validation cohorts. **(B)** The calibration curve of the prediction model in the training cohort. **(C)** The calibration curve of the prediction model in the validation cohort.

The AUC in the training and validation cohorts was 0.910 (0.892–0.929) and 0.894 (0.862–0.925), respectively (**Figure 3A**). There was no significant difference in the diagnostic performance of the prediction model between the two cohorts (*P*=0.379). The optimal cut-off value of the probatility of the prediction model was 26.41%. The result of sensitivity, specificity, PPV, NPV, PLR, and NLR used to distinguish between LCC and CA was 86.4%, 80.1%, 82.2%, 69.1%, 91.9%, 4.342, and 0.170 in the training cohort, as well as 85.1%, 79.1%, 81.1%, 67.0%, 91.4%, 4.07 and 0.188 in the validation cohort, respectively (**Table 4**). The results of the Hosmer–Lemeshow goodness of fit test showed good consistency between the outcome predicted by the model and the actual diagnosis, which was reflected in both the training and validation cohorts (**Figures 3B, C**).

**Table 4:** Predictive performance of the models used to estimate the risk of LCC.

	Training Cohort	Validation Cohort
Area under the curve	0.910	0.894
95% CI lower	0.892	0.862
95% CI upper	0.929	0.925
Sensitivity	86.4%	85.1%
Specificity	80.1%	79.1%
Accuracy	82.2%	81.1%
Positive predictive value	69.1%	67.0%
Negative predictive value	91.9%	91.4%
Positive log-likelihood ratio	4.342	4.07
Negative log-likelihood ratio	0.170	0.188

CI, confidence interval; LCC, localized colorectal cancer.

**Figure 4:**
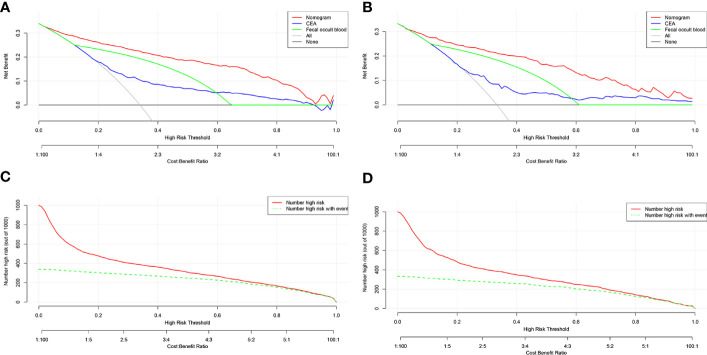
DCA and CIC of the prediction model. **(A)** DCA of the training cohort. **(B)** DCA of the validation cohort. **(C)** CIC of the training cohort. **(D)** CIC of the validation cohort. DCA, decision curve analysis; CIC, clinical impact curve; CEA, carcinoembryonic antigen; FOBT, fecal occult blood test.

When drawing the DCA to reflect the advantages of the new model, we added a comparison between the new model and the two common CRC screening indicators, CEA and FOBT. In the training and validation cohorts, the threshold probability was between 0.05 and 1.00. The performance of the prediction model was better than that of the CEA, FOBT, and two extreme cases (treat-none and treat-all) as shown in **Figures 4A, B**. For example, when the risk threshold was set to 0.3 (i.e. if the LCC probability of the patient was >30%), the patient would receive further treatment. In the training cohort, the net benefit of the new prediction model was 0.25, which was higher than that of of the FOBT (0.20), CEA (0.10), treat-all (0.05), and treat-none (0.00).

CIC results also showed a similar number of high-risk LCC patients predicted by the new model and the actual number of LCC patients in both the training and validation cohorts (**Figures 4C, D**). For instance, when the risk threshold was set to 0.4, almost 400 out of every 1000 persons in the training cohort were deemed at high risk, and approximately 350 of them were diagnosed with LCC.

## 4 Discussion

CA is a benign tumor in the colorectal region, of which only 5% will eventually advance to CRC, and the overall tumor progression is slow (25). Preoperative differentiation between LCC and CA is helpful to reduce unnecessary treatment and promote the early detection of CRC. In most cases, the differentiation between LCC and CA patients depends on invasive colonoscopy (26). In this study, we found that age, sex, drinking history, WBC, lymphocyte count, RDW, albumin, CEA, and FOBT were independent predictors of LCC in patients, and successfully developed a web-based prediction model. Through evaluating calibration and validation, we believe that our prediction model has a good discrimination performance and clinical application value.

To predict LCC and CA, a wide range of variables were considered in this model when selecting preoperative markers. Age and sex play a significant role in the diagnosis of many tumors, including CRC (27) and lung cancer (28). In this study, being female and older were considered risk factors for LCC. Previous studies reported smoking and drinking as risk factors for CRC, and these were associated with a poor prognosis (29–32). The current study also showed that individuals with a drinking history were at a higher risk of being diagnosed with LCC, but there was no association between LCC and smoking. Routine blood cell parameters are often used as inflammatory markers to reflect the patient’s inflammatory immune status. Various studies have shown that higher neutrophil-lymphocyte and platelet-lymphocyte ratios or a high systemic immune inflammatory index (platelet count × neutrophil count/lymphocyte count) were associated with a higher tumor stage, worse differentiation level, and worse prognosis of CRC (33–36). Our prediction model used single blood cell parameters, such as WBC, lymphocyte count, or RDW, instead of the ratio between parameters, which reduces the steps of numerical conversion and simplifies the calculation process. Serum albumin not only reflects the nutritional status of patients but also has a negative correlation with the inflammatory reaction *in vivo*. It is an independent predictor of the prognosis of CRC (37). In this study, patients with lower albumin levels had a higher probability of LCC diagnosis. CEA and FOBT are currently widely used non-invasive markers for screening CRC (12, 38), which played a significant role in this prediction model and had better diagnostic performance than being used individually.

Previous studies attempted to use molecular detection for early diagnosis of CRC; however, this is expensive and incomparable to colonoscopy or fecal immunochemical tests (39–41). Some studies have used markers of the systemic inflammatory response as diagnostic tools for CRC (18, 42). However, the results of these trials have limited diagnostic performance in individuals with early CRC because a large number of patients with advanced CRC were included. Additionally, the static nomograph model requires the manual calculation of the prediction probability corresponding to the total score, which is less intuitive. This study included nine easily available and inexpensive preoperative variables and developed a web-based prediction model. As a preoperative prediction tool, the model is not only easy to popularize and use, but also has a high prediction accuracy and good discrimination characteristics.

Our study has some limitations. First, since this is a single-center retrospective study, it is necessary to validate our model with data from external prospective studies. Second, neither the diagnostic information of patients with advanced CRC nor the prognosis was included in this study. In most cases, the model was mainly used as a tool for early screening of LCC and CA. Third, other indicators with potential predictive value, such as gene expression and coagulation markers, were not included due to restrictions posed by retrospective data as well as the feasibility of sample collection and costs. To validate the accuracy of our findings, a multicenter prospective investigation is required.

## 5 Conclusion

A web-based preoperative prediction model incorporating nine preoperative variables was developed. The model can directly and quantitatively assess the risks and benefits in clinical practice and has strong performance in recognizing LCC and CA.

## Data availability statement

The original contributions presented in the study are included in the article/**Supplementary Material.** Further inquiries can be directed to the corresponding author.

## Ethics statement

The studies involving human participants were reviewed and approved by the Ethics Committee of Dongyang People’s Hospital. Written informed consent for participation was not required for this study in accordance with the national legislation and the institutional requirements.

## Author contributions

YL contributed to the research concept and design. HG and JJ conducted data collection and analysis. All authors participated in the first draft of this manuscript. All authors contributed to the article and approved the submitted version.
