# Supplementary figures and images for: Development and validation of a web-based predictive model for preoperative diagnosis of localized colorectal cancer and colorectal adenoma
Source: Front Oncol. 2023 Aug 17;13:1199868. doi: 10.3389/fonc.2023.1199868 (PMC10470828; doi:10.3389/fonc.2023.1199868)

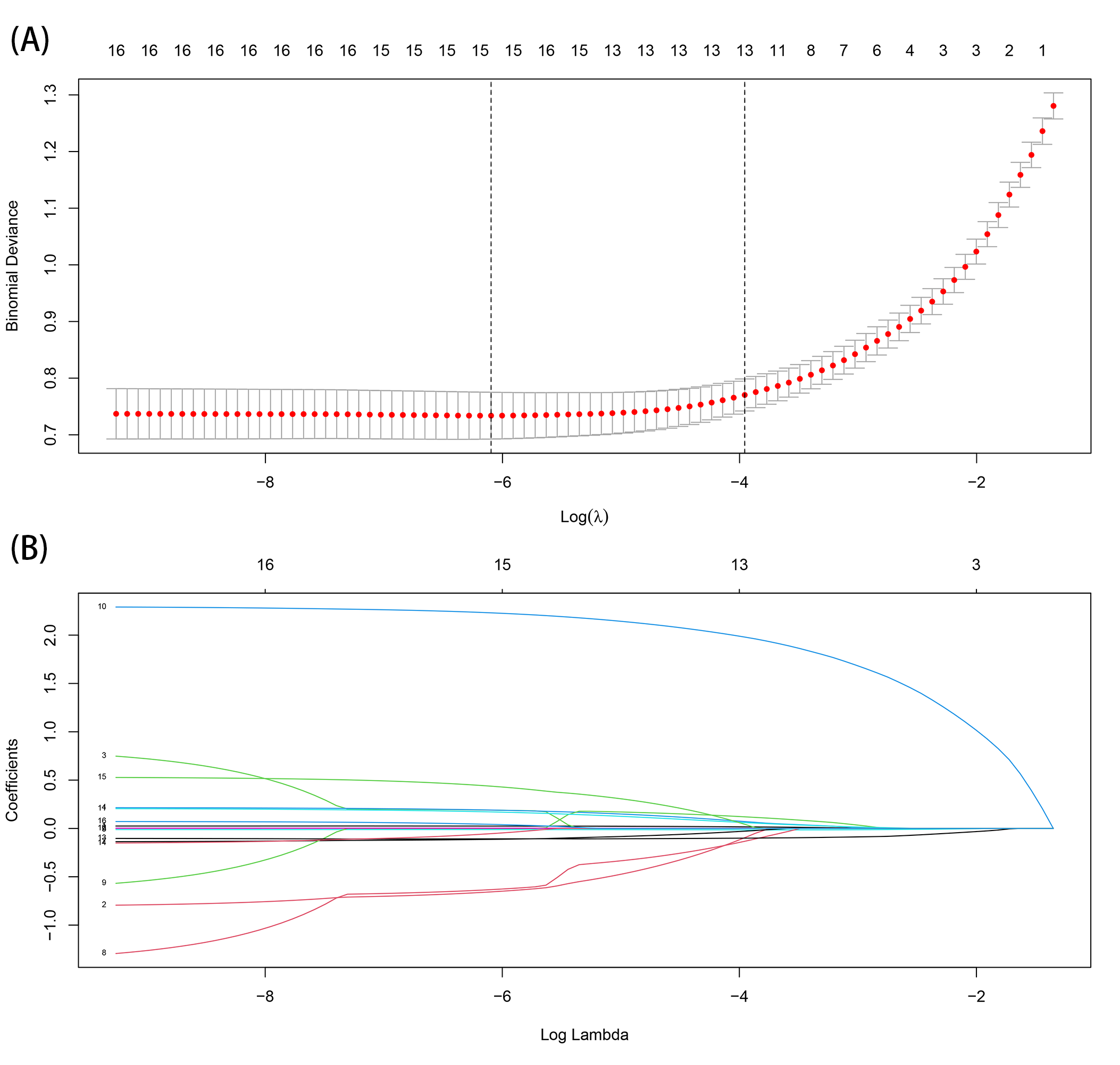

Supplement: Supplementary file 1 [file Image_1.tif]
